# Asparagine biosynthesis as a mechanism of increased host lethality induced by *Serratia marcescens* in simulated microgravity environments

**DOI:** 10.1016/j.heliyon.2022.e09379

**Published:** 2022-05-04

**Authors:** Rachel Gilbert, Nicole Tanenbaum, Sharmila Bhattacharya

**Affiliations:** a NASA Postdoctoral Program, Universities Space Research Association, NASA Ames Research Center, Moffett Field, CA, USA; b Dept. of Biology, University of Pennsylvania, Philadelphia, PA, USA; c NASA Ames Research Center, Moffett Field, CA, USA

**Keywords:** Low shear modeled microgravity, *Drosophila melanogaster*, *Serratia marcescens*, Asparagine, Virulence

## Abstract

While studies have shown an increase in pathogenicity in several microbes during spaceflight and after exposure to simulated microgravity, the mechanisms underlying these changes in phenotype are not understood across different pathogens, particularly in opportunistic pathogens. This study evaluates the mechanism for increased virulence of the opportunistic gram-negative bacterium, *Serratia marcescens,* in simulated microgravity. Low-shear modeled microgravity (LSMMG) is used in ground-based studies to simulate the effects of microgravity as experienced in spaceflight. Our previous findings showed that there was a significant increase in mortality rates of the *Drosophila melanogaster* host when infected with either spaceflight or LSMMG treated *S. marcescens*. Here, we report that LSMMG increases asparagine uptake and synthesis in *S. marcescens* and that the increased host lethality induced by LSMMG bacteria grown in rich media can be recapitulated in minimal media by adding only aspartate and glutamine, the substrates of asparagine biosynthesis. Interestingly, increased bacterial growth rate alone is not sufficient to contribute to maximal host lethality, since the addition of aspartate to minimal media caused an LSMMG-specific increase in bacterial growth rate that is comparable to that induced by the combination of aspartate and glutamine, but this increase in growth does not cause an equivalent rate of host mortality. However, the addition of both aspartate and glutamine cause both an increase in host mortality and an overexpression of asparagine pathway genes in a LSMMG-dependent manner. We also report that L-asparaginase-mediated breakdown of asparagine is an effective countermeasure for the increased host mortality caused by LSMMG-treated bacteria. This investigation underscores the importance of the asparagine utilization pathway by helping uncover molecular mechanisms that underlie increased mortality rates of a model host infected with microgravity-treated *S. marcescens* and provides a potential mitigation strategy.

## 1 Introduction

Human pathogens occupy a wide variety of ecological niches, including those in the harsh conditions imposed by spaceflight. Understanding the effects that spaceflight may have on the adaptation and fitness of human pathogens is necessary to predict potential causes of health problems during future missions, where the availability of treatment and prevention options is limited. This is even more important in the context of opportunistic bacterial pathogens, which typically produce benign infections, until they are challenged by certain environmental conditions or when host immune impairments occur, which can then equip them with the ability to cause serious health concerns [1]. One such environmental condition is spaceflight, which has been demonstrated to convert opportunistic pathogens to primary pathogens, enabling them to take advantage of a compromised immune system that may occur to astronauts during spaceflight [2, 3]. In line with this, there is mounting evidence that primary bacterial pathogens often undergo a diversity of significant phenotypic shifts during spaceflight, including increased antibiotic resistance [4, 5, 6, 7], increased virulence [8, 9, 10, 11], and morphological changes associated with pathogenicity [12]. Despite mounting research in these primary pathogens, much less is known about phenotypic shifts that occur to opportunistic pathogens when exposed to microgravity. Specifically, there is little known about how pathogens may be utilizing nutrients differently in the microgravity environment of space compared to ground microbes, and potentially how these mechanistic changes might be influencing the host immune response during infection.

Due to increased nosocomial reports, the human opportunistic bacteria, *Serratia marcescens,* has emerged as a model for studying rapid shifts in virulence and antibiotic resistance [13, 14]. *S. marcescens* is a ubiquitous pathogen that occupies a wide variety of habitats and hosts. It can adapt quickly to antibiotics independently and simultaneously, which is likely due to the loss of conserved genetic elements and large genomic diversity among strains [14, 15, 16]. Furthermore, *S. marcescens* was found aboard the spacecraft Mir, and in condensed water aboard the International Space Station [17, 18]. Therefore, this ubiquitous pathogen should be monitored closely for spaceflight-induced changes that may pose a threat to immunocompromised astronauts that are more susceptible to opportunistic pathogens and especially considering the complications with antibiotic resistance [2, 18].

Research in our laboratory has demonstrated that there is a significant shift in virulence with *S. marcescens* (Db11) after spaceflight. When cultured aboard the International Space Station, *S. marcescens* Db11 killed healthy ground-reared *Drosophila melanogaster* hosts significantly faster than an identical control of the bacteria that was cultured on the ground [11]. However, the specific mechanisms of increased Db11 virulence and reduced *D. melanogaster* survival are currently unknown. In line with this, increased virulence is observed with various space and low-shear modeled microgravity (LSMMG)-isolated bacteria, including studies with *Salmonella* [19], and *Staphylococcus aureus* [20], which pose a threat to astronaut health during spaceflight. Our prior research has shown that *Serratia marcescens* also shows a significant increase in host lethality of *D. melanogaster* after growth in simulated microgravity (LSMMG) conditions [11].

Since spaceflight studies are costly and can be difficult to perform due to constraints on experimental design and the availability of spaceflight opportunities, many researchers perform ground-based studies using devices such as the rotating wall vessel (RWV) to simulate microgravity [21, 22, 23]. The RWV simulates microgravity by rotating at a speed and orientation which keeps cells suspended in liquid without bubbles, thereby creating a low fluid shear environment that simulates the weightlessness that cells experience during spaceflight [24] and is referred to as the LSMMG treatment. When using the RWV to induce LSMMG, the vessel is rotated such that the axis of rotation is perpendicular to the gravity vector and the rotation offsets the sedimental effects of gravity and keeps the cells in continuous suspension (LSMMG). In contrast, in the control orientation (RWV-Control), the axis of rotation is parallel to the gravity vector and does not offset the sedimentation induced by Earth's gravitational force. Thus, in the RWV-Control, the cells can settle at the bottom of the container and experience Earth gravity (1g) without continuous suspension while acting as a control for any potential biological effects of the vessel itself. Although this method is not an exact recreation of the spaceflight environment, the effects of the LSMMG environment have been demonstrated to recapitulate findings from spaceflight studies [10, 11].

*Drosophila melanogaster*, as used in this study, has proven to be a useful host model for spaceflight and ground-based studies in the past. This is due to their well-characterized innate immune system, tractable genetics, ease of use, short life-cycle, genetic homology to mammalian systems, and low resource requirements for breeding large colonies [11, 25, 26, 27, 28, 29, 30, 31, 32, 33, 34, 35].

In the current study, we use LSMMG to investigate the specific mechanisms that enable microgravity-treated *Serratia marcescens* bacteria to kill the host *D. melanogaster* more quickly than control bacterial cultures. A screen of several genes related to known *S. marcescens* virulence factors did not reveal any differences of expression in LSMMG compared to RWV-Control. Further testing revealed a LSMMG-dependent overexpression of the gene asparagine synthetase B (*asnB*), which is implicated in virulence and resistance to drugs in other pathogenic bacteria [36, 37]. This gene is involved with amino acid metabolism and has been linked to both pathogen growth and virulence [38]. Amino acids like asparagine are thought to be important to the competition between hosts and pathogens in determining the outcome of an infection [38]. Since our previous study indicated that the LSMMG-reared *S. marcescens* killed the host faster after infection [11] than control *S. marcescens* cultures, we wanted to explore whether the asparagine pathway might be linked to the increased host mortality caused by LSMMG treatment.

Here, we show that LSMMG treatment causes a change in the expression of key asparagine pathway related genes in *S. marcescens* with a concomitant increase in host mortality. Further, we find that these changes in key asparagine pathway genes and increased host mortality can be recapitulated in an LSMMG-specific manner by the addition of aspartate and glutamine (the substrates of asparagine synthesis) to minimal media. We also show that the addition of L-asparaginase, which catabolizes asparagine, can mitigate the LSMMG-induced increase in host virulence. Thus, this study allows us to understand the phenotypic shifts occurring in microbes exposed to altered gravity, and more specifically, how amino acid metabolism may affect the mortality rate of the host. In addition, these results will allow us to explore the asparagine pathway further and develop countermeasures that target amino acid biosynthesis in opportunistic pathogens such as *S. marcescens, as* an essential consideration to maintain host health during spaceflight.

## 2 Results

### 2.1 The asparagine pathway and extracellular asparagine and glutamine concentrations are affected by simulated microgravity treatment of *S. marcescens*

**Figure 1:**
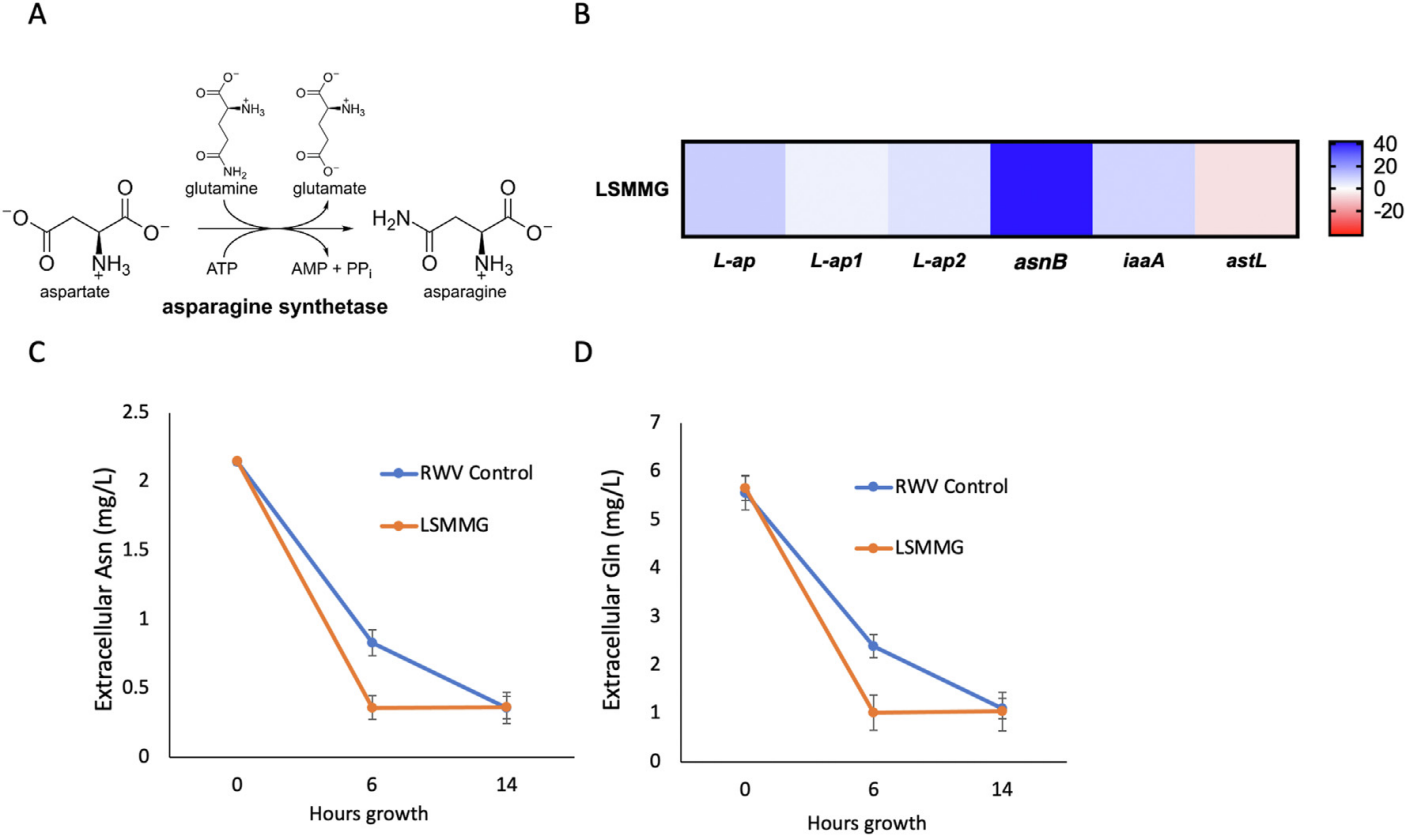
The asparagine pathway and increased uptake of extracellular asparagine and glutamine from nutrient-rich LB media under simulated microgravity. Measurements of amino acid consumption were performed by removing cells from media by centrifugation and utilizing spectrophotometric analysis with Megazyme™ reagent. A) The asparagine biosynthesis pathway. B) At the end of the growth period of *S. marcescens* in simulated microgravity (LSMMG orientation), gene expression for each sample was measured via qPCR: *L-ap* (L-asparaginase), *L-ap1* (L-asparaginase 1), *L-ap2* (L-asparaginase 2), *asnB* (asparagine synthetase B), *iaaA* (isoaspartyl peptidase/L-asparaginase), or *astL* (asparagine tRNA ligase). Expression values are reported as fold change relative to the RWV-Control orientation and calculated using the ΔΔ Ct method. Specific genes represented by the blue end of the color spectrum indicated gene overexpression and those towards the red end indicated inhibition of gene expression. Error bars represent one standard error. Measurements of C) extracellular asparagine and D) extracellular glutamine were performed at hours 0, 6, and 14 of growth in the rotating wall vessel, with an initial starting concentration of 1 × 10^8^ CFU.

Our previous findings have indicated that *S. marcescens* exposed to LSMMG for 24 h grow at a faster rate inside the host after infection and caused increased host lethality in *D.melanogaster* [11]. First, we looked at several genes involved in flagella formation, membrane-bound proteins, and known virulence genes, and did not see significant upregulation in LSMMG compared to the control bacteria (Table 3). We, therefore, looked at other genes linked to bacterial growth mechanisms and found that genes related to asparagine metabolism were upregulated in LSMMG. More specifically, we found that there was a significant upregulation in *asnB* (asparagine synthetase B), which is a gene that regulates the conversion of aspartate to asparagine using ATP and glutamine (Figure 1A,B). Therefore, we evaluated the expression of genes that are important for asparagine and glutamine metabolism. Gene expression analyses showed that the expression level of the gene *asnB* (asparagine synthetase B) was 39.17 fold higher in LSMMG-treated cells relative to the RWV-control (P < 0.0001), as well as a 10.5 fold increase in expression of L-asparaginase (*L-ap*, P = 0.054), but there was no significant difference in expression for the other genes tested, although several genes showed a positive trend for expression levels: *L-ap1* (L-asparaginase 1), *L-ap2* (L-asparaginase 2), *iaaA* (isoaspartyl peptidase/L-asparaginase), or *astL* (asparagine tRNA ligase) (Figure 1D). *asnB* is a gene that catalyzes the conversion of aspartate to asparagine using ATP and glutamine, and is known to be linked to virulence and antibiotic resistance in bacterial pathogens [36, 37]. The L-asparaginase subunits assist with asparagine catabolism and nitrogen homeostasis [39], therefore, an overexpression of these genes suggests that there is an increase in asparagine catabolism as part of the overall homeostatic regulation of the asparagine pathway.

The observation that *asnB* expression is significantly increased, led us to hypothesize that there is an increased presence of asparagine in LSMMG that may contribute to the increase in pathogen growth rate, and therefore an increase in consumption of glutamine and aspartate in the media. To confirm this, we quantified the rates at which *S. marcescens* utilized extracellular glutamine and asparagine in the nutrient-rich LB media, in order to see whether the consumption rates of these amino acids matched the increased growth of the bacteria in LSMMG. For asparagine measurements (Figure 1C), there was no difference between the RWV-control and the LSMMG at 0 h (F = 4.348, P = 0.11) or at 14 h (F = 0.007, P = 0.939). At 6 h of growth, LSMMG had significantly lower extracellular asparagine than the RWV-Control (F = 1540.68, P < 0.0001). Similarly, extracellular glutamine (Figure 1D) was not significantly different at hours 0 (F = 2.251, P = 0.208) or 14 (F = 0.669, P = 0.459), but LSMMG had significantly lower glutamine at hour 6 (F = 282.056, P < 0.0001). These results suggest that the *S. marcescens* in LSMMG are consuming extracellular glutamine and asparagine more quickly during the active growth phase of the culture, which is consistent with the increase in growth that we see in LSMMG-reared bacteria in rich media [11].

Taken together, the almost 40-fold overexpression of the *asnB* gene in LSMMG coupled with the overexpression of the asparaginase subunits (Figure 1B), along with the increased consumption of asparagine (Figure 1C), indicated that the asparagine pathway was likely to play a significant role in the phenotypic changes observed in *S. marcescens* under reduced gravity LSMMG conditions.

### 2.2 *S. marcescens* growth is affected by L-asparaginase treatment in vitro under simulated microgravity conditions

**Figure 2:**
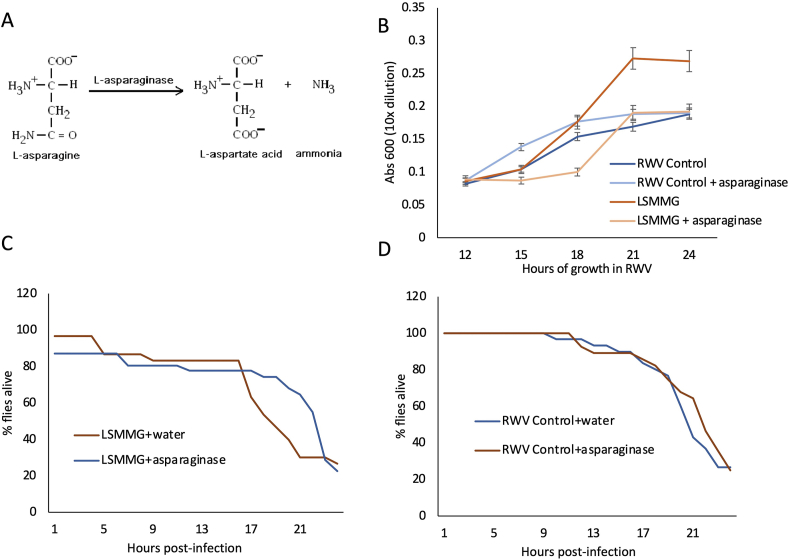
Exogenously added L-asparaginase can inhibit growth of LSMMG-treated bacteria and reduce host-lethality. A) The breakdown of asparagine catalyzed by the asparaginase enzyme. B) The starting concentration of 1 × 10^8^ CFU of *S. marcescens* was placed in a 10 mL rotating wall vessel at 37 °C in LB media. One hundred microliters of L-asparaginase was added to the growth media, and growth of bacteria estimated by absorbance at 600 nm was measured every 3 h. L-asparaginase addition in vitro reduces the rate of growth most significantly in the LSMMG samples. Error bars represent one standard error. C and D) Bacteria were grown in RWV with LB media only. After 24 h of growth, bacteria were fixed in 20% glycerol for injections. Flies were injected with *S. marcescens*. One hour after infection, flies were then injected with a treatment of L-asparaginase, or sterile water (no treatment). The LSMMG bacteria kills flies at a lower rate when L-asparaginase is injected in vivo (C) and similarly in vivo injected L-asparaginase does not impact survival of the RWV-Control (D). (The statistical results for the comparisons shown in Figures 2C and 2D are included in Table 1.)

Since we saw an increase in asparagine consumption with LSMMG treatment, we studied whether the depletion of exogenous L-asparagine using the enzyme L-asparaginase would negatively influence growth in LSMMG. When *S. marcescens* was grown in LSMMG with asparaginase, growth was significantly lower than the LSMMG without asparaginase at hours 18 (P = 0.034), 21 (P = 0.0004), and 24 (P = 0.0008) of growth (Figure 2B). For the RWV-Control sample, asparaginase reduced growth only at hour 15 (P = 0.031), but not at any other timepoints. The LSMMG + asparaginase growth was significantly lower than the RWV-Control at hour 18 (P = 0.0296) but was not significantly different from the control at any other time point (Figure 2B). These results provide evidence that L-asparaginase reduces the growth of *S. marcescens* in the LSMMG condition compared to the control, but does not significantly impact growth in the control samples, suggesting that the growth-suppressive effect of L-asparaginase is LSMMG-specific.

**Table 1:** Results of the proportional hazards survival model for the asparaginase treatment injections (Figure 2). ‘Ratio’ refers to the ratio of the hazard rates corresponding to the conditions described by Level 1 and Level 2, respectively. This value can be interpreted as a magnitude of differences in survival between Level 1 and Level 2. Hours 1–24 refers to the full analysis of all 24 data points for the survival experiment, 24 h total. Hours 12–24 refers to the final 12 h of the experiment, which is when the majority of the death in the survival analysis occurs. This distinction is made due to the effects of the treatment being applied in this experiment, which has the strongest effect during this time period.

Level1	Level2	Risk Ratio	Prob > Chisq
**Hours 1–24 Post-infection**			
1g-water	1g-asparaginase	1.3352328	0.3485
LSMMG-asparaginase	1g-asparaginase	5.5078408	<.0001∗
LSMMG-asparaginase	1g-water	4.125004	<.0001∗
LSMMG-water	1g-asparaginase	5.5609204	<.0001∗
LSMMG-water	1g-water	4.1647571	<.0001∗
LSMMG-water	LSMMG-asparaginase	1.0096371	0.9766
**Hours 12–24 Post-infection**			
1g-water	1g-asparaginase	1.4371124	0.3784
LSMMG-asparaginase	1g-asparaginase	1.4095858	0.4001
LSMMG-water	1g-asparaginase	4.1156768	0.0018∗
LSMMG-water	1g-water	2.8638518	0.0134∗
LSMMG-water	LSMMG-asparaginase	2.9197774	0.0197∗
1g-water	LSMMG-asparaginase	1.0195281	0.9627

### 2.3 Injection of LSMMG *S. marcescens* with in vivo L-asparaginase treatment reduces lethality in the *D. melanogaster* host

Next, since the synthesis of asparagine by *S. marcescens* was closely linked with LSMMG treatment, we investigated whether L-asparaginase could mitigate increased virulence of LSMMG-reared pathogens after injection into a host as measured by host mortality. To do this, LSMMG or RWV-Control-treated *S. marcescens* were injected into *D. melanogaster* hosts, and the infected hosts were then injected again with L-asparaginase 1 h after bacterial infection. There was no difference in overall fly survival between the LSMMG and the LSMMG + asparaginase treatment in the early part of the survival curve indicating that there was no harmful effect from asparaginase injections of the host (Figure 2C, P = 0.0976). However, when the survival curve was analyzed for the latter half of the experiment (12–24 h) when the majority of the host death typically occurs from an *S. marcescens* infection, then there was a significant difference in survival with and without asparaginase treatment. We demonstrated that the LSMMG-injected flies died faster than the LSMMG with asparaginase treatment (Figure 2C, Risk Ratio = 2.92, P = 0.0197). There was no difference between the RWV-Control with water or with asparaginase (Figure 2D, Risk Ratio = 1.33, P = 0.348). For the complete result of this statistical analysis, please see Table 1. These results indicate that the addition of exogenous asparaginase into the host, during infection with *S. marcescens,* can inhibit the increased *S. marcescens* virulence that is associated with reduced gravity treatment of the microbe (Figure 2C). L-asparaginase does not prevent host death and does not affect the time-course of host lethality in the RWV-Control bacteria (Figure 2D), but it does target the gravity-specific increase in host lethality by *S. marcescens* that we have shown in our previous study [11].

### 2.4 Both aspartate and glutamine supplementation in combination are required in minimal media to recapitulate the asparagine pathway gene expression changes seen in rich media

**Figure 3:**
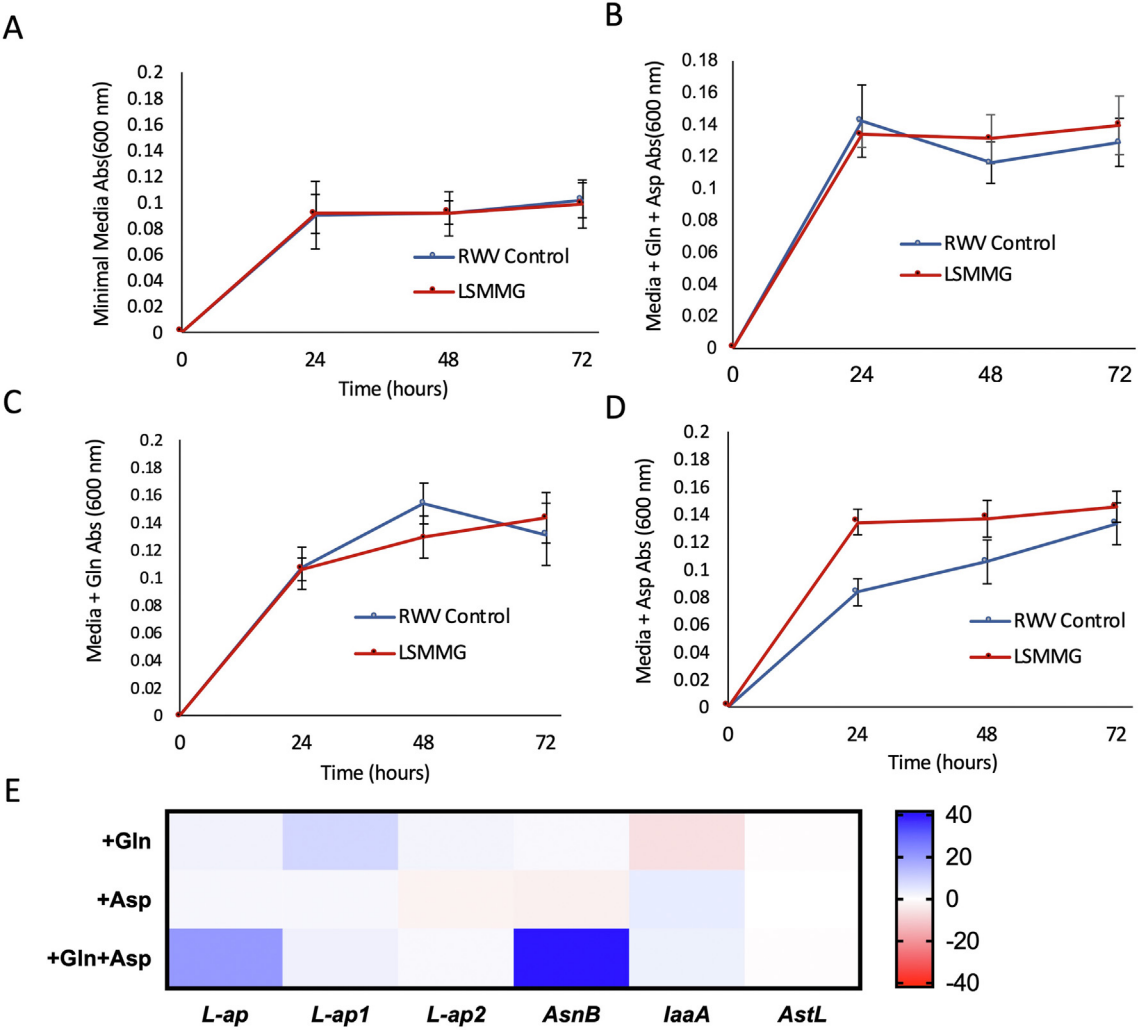
Growth and gene expression changes of LSMMG-treated *S. marcescens* in Davis minimal media supplemented with amino acids. Starting concentration of 1 × 10^8^ CFU of *S. marcescens* was placed in a 10 mL rotating wall vessel at 37 °C, containing A) Davis minimal media only, B) Davis minimal media supplemented with 10 mM aspartate and 10 mM glutamine, C) Davis minimal media supplemented with 10 mM glutamine, and D) Davis minimal media supplemented with 10 mM aspartate. Growth was measured every 24 h for 72 h. E) At the end of growth period, gene expression for each sample was measured via qPCR:: *L-ap* (L-asparaginase), L-ap1 (L-asparaginase 1), *L-ap2* (L-asparaginase 2), *asnB* (asparagine synthetase B), *iaaA* (isoaspartyl peptidase/L-asparaginase), or *astL* (asparagine tRNA ligase). Expression values are reported as fold change in LSMMG relative to the Davis minimal media values and calculated using the ΔΔ Ct method. Even though aspartate addition increases growth in LSMMG cultures but *asnB* and *L-ap* genes are overexpressed in LSMMG compared to control only when both glutamine and aspartate are present.

Next, we examined the effect of specific amino acids that are substrates in the asparagine synthesis pathway, on bacterial growth kinetics and gene expression. To do this, we grew *S. marcescens* in a minimal media supplemented with glutamine, aspartate, or both amino acids together, in LSMMG and in the RWV-Control. For all amino acid growth measures, there was no significant difference between the LSMMG and RWV-Control *S. marcescens*, except for the aspartate-supplemented sample at 24 h (Figure 3D, F = 1.76, P = 0.0023). This suggests that aspartate in isolation is sufficient to stimulate the LSMMG-specific increase in growth.

However, when we looked at how these growth conditions influenced gene expression, the expression level of 38.95 relative to the RWV-control was significantly higher for the gene *asnB* (asparagine synthetase *B*) in the combined glutamine and aspartate supplemented media but not in the media with either glutamine or aspartate alone (Figure 3E, P < 0.0001). Similarly, the gene *L-ap* (L-asparaginase) with a relative expression value of 21.45 was significantly higher in the combined media than in the glutamine or aspartate media alone (P < 0.0001). There was no significant difference across media conditions for the other genes tested: *L-ap1* (L-asparaginase 1), *L-ap2* (L-asparaginase 2), *iaaA* (isoaspartyl peptidase/L-asparaginase), or *astL* (asparagine tRNA ligase). This suggests that while aspartate alone can increase the growth of *S. marcescens* in LSMMG in minimal media, the presence of both aspartate and glutamine are necessary to induce the LSMMG-specific overexpression of *asnB* at a similar level to the nutrient-rich media (Figure 1B and Figure 3E).

### 2.5 Maximal host mortality rates of *D. melanogaster* are seen only when minimal media is supplemented with aspartate and glutamine, substrates of asparagine synthesis

**Figure 4:**
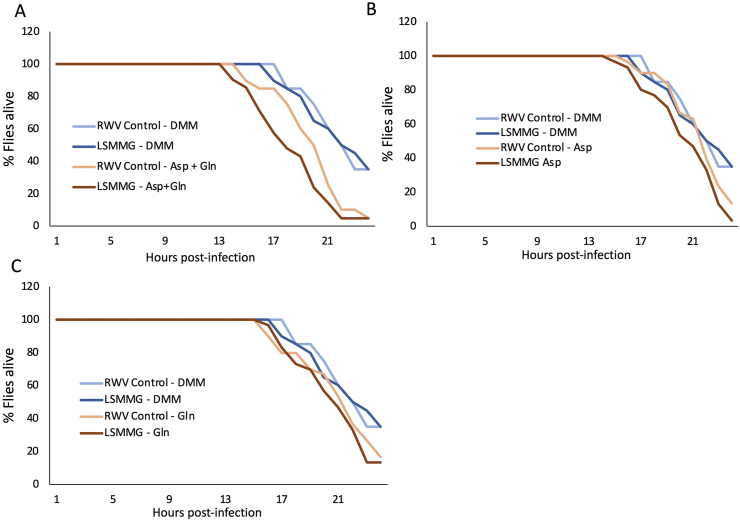
Infection of flies with *S. marcescens* shows maximum host mortality with LSMMG bacteria when supplemented with both aspartate and glutamine. Sample was grown in A) Davis minimal media (DMM) either alone or with 10 mM aspartate and 10 mM glutamine, B) Davis minimal media supplemented with 10 mM aspartate, C) Davis minimal media supplemented with 10 mM glutamine. Bacteria were grown for 24 h in the RWV, then fixed in 20% glycerol for injections into *D. melanogaster* hosts. Both glutamine and aspartate must be present together to elicit the maximum LSMMG-induced increase in host mortality as shown in Figure 4A. (The full statistical results for all comparisons can be seen in Table 2.)

Given the significantly increased expression of asnB in LSMMG-grown bacteria in minimal media supplemented with both aspartate and glutamine, it was important to test whether bacteria grown in this way would also increase its virulence in the *D. melanogaster* host. In the minimal media supplemented with both aspartate and glutamine, flies injected with the bacteria grown in LSMMG died faster than those injected with bacteria grown in the RWV-Control (Ratio = 2.46, P = 0.0021, Figure 4A). Similarly, the media with aspartate supplementation showed an increase in the rate of fly death after injection with the LSMMG-grown bacteria compared to the control (Ratio = 2.26, P = 0.007, Figure 4B). For the LSMMG-grown bacteria, there was a greater rate of fly death from the bacteria grown in aspartate and glutamine media compared to the bacteria grown in aspartate alone (Ratio = 1.98, P = 0.0221, Figure 4A,B). There is no significant difference in mortality rates between the flies injected with bacteria reared in LSMMG with a media composition composed of just the Davis minimal media (P = 0.46) or in the minimal media with just glutamine added (P = 0.55, Figure 4C). The full results of these comparisons can be seen in Table 2. This indicates that while either aspartate alone or both aspartate and glutamine together, can cause an increase in bacterial growth rate in vitro, only the combination of the two amino acids together can cause the LSMMG-specific induction of *asnB* expression and also causes the maximum increase in LSMMG-dependent virulence of the bacteria in terms of mortality of the fly host (Figures 3 and 4). In other words, the increased growth rate in the LSMMG is not a complete determinant for the increased virulence of *S. marcescens*, but that a change in the metabolism of nutrients like asparagine plays a critical role in the heightened mortality induced by gravity-treated bacteria on a host.

**Table 2:** Results of the proportional hazards survival model for the minimal media supplementation injections (Figure 4). ‘Ratio’ refers to the ratio of the hazard rates corresponding to the conditions described by Level 1 and Level 2, respectively. This value can be interpreted as a magnitude of differences in survival between Level 1 and Level 2.

Level1	/Level2	Risk Ratio	Prob > Chisq
LSMMG DMM	1g DMM	1.2503237	0.4608
LSMMG Asp + Gln	1g Asp + Gln	2.4616875	0.0021∗
LSMMG Asp + Gln	1G Asp	4.485328	<.0001∗
LSMMG Asp + Gln	1g DMM	7.3218004	<.0001∗
LSMMG Asp + Gln	1G Gln	2.7200956	0.0010∗
LSMMG Asp + Gln	LSMMG Asp	1.9824812	0.0221∗
LSMMG Asp + Gln	LSMMG DMM	5.8559238	<.0001∗
LSMMG Asp + Gln	LSMMG Gln	2.2930899	0.0056∗
LSMMG Asp	1G Asp	2.262482	0.0078∗
LSMMG Asp	1g DMM	3.6932508	<.0001∗
LSMMG Asp	1G Gln	1.3720662	0.2776
LSMMG Asp	LSMMG DMM	2.9538357	0.0005∗
LSMMG Asp	LSMMG Gln	1.1566767	0.615
LSMMG Gln	1G Asp	1.9560193	0.0278∗
LSMMG Gln	1g DMM	3.1929845	0.0003∗
LSMMG Gln	1G Gln	1.1862141	0.5564
LSMMG Gln	LSMMG DMM	2.5537263	0.0022∗
1g Asp + Gln	1G Asp	3.0685957	0.0004∗
1g Asp + Gln	1g DMM	5.0091421	<.0001∗
1g Asp + Gln	1G Gln	1.8609283	0.0365∗
1g Asp + Gln	LSMMG Asp	1.3562962	0.2974
1g Asp + Gln	LSMMG DMM	4.0062762	<.0001∗
1g Asp + Gln	LSMMG Gln	1.5687963	0.1245
1G Asp	1g DMM	1.6323891	0.1061
1G Asp	LSMMG DMM	1.3055732	0.377
1G Gln	1G Asp	1.6489597	0.0953
1G Gln	1g DMM	2.6917438	0.0015∗
1G Gln	LSMMG DMM	2.1528375	0.0113∗

## 3 Discussion

In this study, we provide evidence for the importance of nutrient utilization during bacterial growth as a factor for increased host mortality after pathogen growth in LSMMG. Host mortality is a direct measure of pathogen virulence, and therefore an increased rate of host death is one indication of increased virulence of a pathogen [40]. While there are studies showing an increase in virulence of pathogens after exposure to spaceflight and simulated microgravity, the specific mechanisms that underlie these changes are largely unknown. In our previous study [11], we found that *S. marcescens* in spaceflight and in LSMMG show an increase in virulence via increased rate of host mortality, and that the *in vivo* growth of the pathogen after infection of the host is higher from both spaceflight and LSMMG treated bacteria. While *in vivo* growth is only an indirect proxy for virulence, faster pathogen growth immediately following infection is often linked to the ability of the pathogen to overcome host immunity and sustain infection [41, 42]. Therefore it was necessary for us to examine in more detail the specific potential mechanisms for increased virulence of *S. marcescens* in LSMMG.

First we looked at several genes involved in flagella formation, membrane-bound proteins, and known virulence genes, and did not see significant upregulation in LSMMG compared to the control (Table 3). We therefore looked at other genes linked to bacterial growth mechanisms, and found that genes related to amino acid metabolism were upregulated in LSMMG (Figure 1B). We found that several genes within the asparagine synthesis pathway were overexpressed in LSMMG, but specifically saw the largest overexpression, an almost 40-fold increase, in the asparagine synthetase B (*asnB* gene). Asparagine synthetase B is an enzyme that catalyzes the synthesis of asparagine using aspartate, and glutamine as a nitrogen source [43, 44]. The deletion of the *asnB* gene leads to a slow-growth phenotype in the pathogen *Bacillus subtilis*, and in most pathogens (including *S. marcescens*) is the main gene involved in asparagine biosynthesis [45]. This gene has not been implicated previously in *S. marcescens* virulence, but has been shown to mediate virulence and resistance to drugs in other bacterial pathogens [36, 37]. While this gene and others in amino acid uptake pathways are important for growth and division of the cell, they are also important mediators of host-pathogen interactions [38]. While the host utilizes amino acid metabolism to support defense responses against a pathogen, conversely, the pathogen can modulate amino acid metabolism for its own advantage [38]. Therefore, amino acid utilization and assimilation mechanisms can be important mediators of infection at the host-pathogen interface, and asparagine metabolism has been linked to virulence and resistance of some pathogens to antibiotics [36, 37, 38].

**Table 3:** Expression values of candidate genes in *S. marcescens* for growth, motility, and virulence as measured by qPCR. Expression values are reported as fold change in LSMMG relative to RWV-Control and calculated using the ΔΔ Ct method. Samples were grown in the RWV for 24 h, then fixed in RNAlater for extraction. Samples were run with three technical replicates per plate, and were performed in three biological replicates. Values reported are the average across all replicates. All averaged replicates reported are p < 0.01.

Gene Name	Fold Change
FlgG	2.09
secY	2.06
FliE	2.05
nudE	2.00
IpxD	2.04
tatB	2.08
hslU	2.31
rseA	0.88
groEL	1.21
dnaK	2.00
dinF	-0.5

In this study we characterize the underlying indicators for the increased host mortality that we previously reported after infection with microgravity-treated *S. marcescens* [11]. We show that *S. marcescens* grown in LSMMG consume key amino acids glutamine and asparagine at a faster rate than the control (Figure 1C, D), suggesting that the LSMMG might be priming these pathogens to compete with responding host immune cells for extracellular amino acids necessary for proliferation. In fact, this is the method by which some pathogens are successful colonizers of their hosts. The virulence of pathogen *Francisella tularensis* is dependent on the pathogen's ability to replicate inside the host and escape from macrophages [46]. Asparagine acquisition is critical in this pathogen's success in replicating inside the host cell, where they use the host as a substrate for asparagine and aspartate necessary to replicate [46]. Similarly, *S. marcescens* has been shown to have the ability to survive and proliferate inside non-phagocytic host cells, suggesting that there may be a similar mechanism of host-pathogen competition for amino acids [47]. We see in our study that growth in LSMMG increases the expression of *asnB* (Figure 1B), implying an increased consumption of aspartate and glutamine to synthesize asparagine more efficiently, that may allow these pathogens to out-compete host immune cells for amino acids during infection. The presence of both aspartate and glutamine causes a significant overexpression of *asnB*, even when they are the only amino acids present in minimal media (Figure 3E). This suggests that these two amino acid substrates, which are both required for asparagine synthesis, are important for LSMMG-induced overexpression of *asnB*. Therefore, we believe that the LSMMG-induced increase in pathogen virulence may be mediated by the ability of *S. marcescens* to utilize asparagine more efficiently within the host after infection, although further studies are needed to evaluate how *S. marcescens* reared in LSMMG are specifically interacting with the host's immune cells.

We also found evidence that there is an overexpression of some L-asparaginase subunits, including L-asparaginase (Figure 3E and Figure 1B) when *asnB* is overexpressed in the presence of its substrates, aspartate and glutamine. The homeostatic maintenance of the asparagine pathway consists of the anabolic and catabolic processes catalyzed by asnB and L-ap respectively which could result in the upregulation of both enzymes with increased asparagine metabolism. In *Salmonella typhimurium* infections, it has been shown that L-asparaginase suppresses T-cell responses by suppressing T-cell blastogenesis, proliferation, and cytokine production in the host, and that this effect could be attenuated by treatment with L-asparagine [48]. This highlights the fact that asparagine can be a key amino acid both for mammalian immune responses to bacterial pathogens, and is also important for bacterial growth and virulence properties. In this study, we provide evidence that the asparagine pathway is a critical determinant of the LSMMG-dependent increase in host mortality induced by *S. marcescens*.

While aspartate alone can increase *in vitro* growth of *S. marcescens* in an LSMMG-specific manner (Figure 3D), the combination of both aspartate and glutamine (substrates for asparagine synthesis) are required to cause the maximal increase in host lethality during the infection of *D.melanogaster* with LSMMG treated bacteria (Figure 4A). Similarly, the presence of both aspartate and glutamine are required to elicit the full induction of *asnB* gene expression which is not seen with aspartate or glutamine addition alone (Figure 3E). Interestingly, these results indicate that increased division rate of the pathogen during the growth phase alone (Figure 3) is not sufficient to recapitulate the full extent of host mortality observed after infection with microgravity-treated *S. marcescens* (Figure 4). Therefore, the microgravity dependent increase in host mortality induced by *S. marcescens*, appears to be linked specifically with asparagine pathway function under LSMMG conditions, and cannot be fully accounted for simply by increasing bacterial growth rate with the addition of amino acid substrates. To further characterize the effect of the asparagine pathway, the direct suppression of the expression of *asnB* through controlled genetic mechanisms was not within the scope of this study. However, as mentioned above, we demonstrated that the attenuation of the asparagine pathway, via the absence of relevant amino acids from the growth media, suppresses the overexpression of *asnB* in LSMMG (Figure 3E) and correlates with increased host survival (Figure 4B,C). Additional studies that target *asnB* gene with a knockdown will be conducted in future.

When we co-infect flies with both LSMMG-reared *S. marcescens* and exogenously-added purified L-asparaginase, we see a therapeutic effect, and the flies die at a significantly slower rate than the LSMMG-reared bacteria injected alone (Figure 2C). This is in line with our hypothesis that growth in LSMMG is priming *S. marcescens* with the ability to compete effectively with the host for asparagine utilization. The addition of L-asparaginase would break down extracellular asparagine, thereby diminishing the ability of the bacteria to out-compete the host's ability to utilize asparagine for its cellular processes. Consistent with this, L-asparaginase added to the growth medium causes suppression of *S. marcescens* growth, but only in LSMMG and not in the RWV-Control (Figure 2B). These results suggest that additional exogenous L-asparaginase has a detrimental effect on the pathogen itself and causes it to become less virulent in LSMMG, perhaps by reducing the amount of available asparagine that otherwise would have enhanced the LSMMG-treated microbe's ability to utilize this amino acid in the host's body. In growth media without the presence of exogenous L-asparaginase, the L-asparagine in the growth media is consumed at a significantly faster rate in LSMMG than in the RWV-Control (Figure 1C), and a similar effect is seen with the consumption of glutamine (Figure 1D). In some pathogens, mutants lacking the ability to synthesize glutamine or to acquire glutamine from the host, show attenuated virulence, since this is an important source of nitrogen assimilation during colonization of the pathogen in the host [49, 50]. These studies further support the idea that amino acids that are critical for several cellular processes, including protein synthesis, can also be important for pathogen virulence.

To summarize the results of our study, we report that genes related to asparagine biosynthesis were overexpressed after exposing *S. marcescens* to LSMMG, and that the consumption rates of extracellular asparagine and glutamine in rich media were higher in LSMMG than in the control (Figure 1). In nutrient-limited media, these gene expression and virulence patterns can be recapitulated by supplying only the precursors for asparagine biosynthesis: glutamine and aspartate (Figures 3E and 4A). The addition of aspartate causes a LSMMG-specific increase in growth of *S. marcescens* but not a concomitant increase in *asnB* gene expression (Figure 3D,E). Therefore, LSMMG-specific increase in expression of the *asnB* gene is only seen in media that contains both aspartate and glutamine, the precursors for asparagine synthesis (Figure 3E). Consistent with this, the addition of both aspartate and glutamine in the media during growth is also necessary for the greatest increase in host mortality in LSMMG compared to the control (Figure 4A). Furthermore, the coinjection of exogenous asparaginase during the infection of fly hosts with LSMMG-reared *S. marcescens,* results in a therapeutic effect and the flies die more slowly than with injections of the LSMMG-treated bacteria alone (Figure 2C).

The results of this study provide compelling evidence for the involvement of the asparagine pathway in increasing *S. marcescens'* effectiveness to kill its host in a microgravity-specific manner. This study provides a novel mechanism by which microgravity might be affecting *S. marcescens* virulence via modified nutrient utilization capabilities. Importantly, we found that L-asparaginase could be used as a potential therapeutic agent during infections with bacteria in LSMMG. An interesting sequelae to this study would be to test the L-asparaginase as a countermeasure where both the host and the pathogen are exposed to microgravity during spaceflight. This study also furthers our need for understanding how LSMMG may be a unique stressor on bacterial pathogens, and emphasizes the importance of the use of ground-based models in informing spaceflight research.

### 3.1 Limitations of the study

While this work provides evidence that LSMMG is priming the pathogen to out-compete the host immune system by the efficient utilization of amino acids such as asparagine, we have not yet evaluated the metabolomics of the host-pathogen interface after infection. That data will be collected in a subsequent study as a follow-on to this body of work where we have identified a novel mechanistic pathway that correlates *Serratia marcescens* virulence induced by reduced gravity environments. We were also constrained to using qPCR as the method for analyzing gene expression in this study, although RNA-seq could provide a more global assessment of gene expression changes. However, we have RNA-seq analyses ongoing for *S. marcescens* samples from a recent spaceflight mission. We anticipate that RNA-seq data will provide additional data to query the LSMMG-treated bacterial samples for additional genetic pathways that may be involved, since both spaceflight and LSMMG treatment results in increased growth and host mortality induced by *S. marcescens*. Lastly, while *D. melanogaster* is an excellent model for characterizing host-pathogen interactions, a future goal would be to conduct complementary studies in mammalian models in preparation for testing countermeasures like asparaginase for future spaceflight experiments.

### 3.2 Resource availability

The datasets generated during and/or analyzed during the current study are available from the corresponding author on request.

## 4 Methods

### 4.1 *D. melanogaster* fly strains

Fly lines were maintained in a 12-hour light-dark cycle on cornmeal-agar media (torula yeast, dextrose, cornmeal, agar, Tegosept, propionic acid, ethanol) at ambient temperature (∼24 °C). Fly line used in this study was obtained from the Bloomington Stock Center, *w*^1118^ (#3605).

### 4.2 Growth of *S. marcescens* Db11 in rotating wall vessel (RWV)

*S. marcescens* Db11 were taken from stock cultures frozen at -80 °C in Copan Cryovials and were grown in sterile 50 mL conical tubes at 37 °C in liquid LB media containing 100 μg/mL streptomycin for 24 h. Liquid subcultures were then diluted down to A600 of 0.100 in LB media and loaded into a sterile 10 mL rotating wall vessel (Synthecon). The RWV simulates microgravity by rotating at a speed and orientation which keeps cells suspended in liquid without bubbles and simulates the weightlessness that cells experience during spaceflight [24] and this is referred to as the LSMMG treatment. When using the RWV to induce LSMMG, the vessel is rotated with an axis of rotation that is perpendicular to the orientation of the gravity vector and the rotation offsets the sedimental effects of gravity and keeps the cells in continuous suspension (LSMMG). In contrast, in the control orientation (RWV-Control), the axis of rotation is parallel to the gravity vector and does not offset the sedimentation induced by Earth's gravitational force. Thus, in the RWV-Control, the cells can settle at the bottom of the container and experience Earth gravity (1g), thereby acting as a control for any potential biological effects of the container vessel itself [19]. Air bubbles were removed from the vessel and vessels were sealed. Pairs of RWV were then placed in either vertical (simulated microgravity) or horizontal (normal gravity, or RWV-Control) orientation at 37 °C and rotated at 25 rpm (as in Nickerson et al. [19]). Samples were grown for 24 h to ensure that stationary phase was reached.

### 4.3 Asparagine and glutamine quantification

At 6 and 14 h of growth, 200 μl of sample was taken from the RWV using a sterile 2 mL syringe, and the sample was used per manufacturer protocol to estimate abundances of L-asparagine and L-glutamine. The sample removed from the RWV was replaced with sterile PBS, and any air bubbles were removed before resuming rotation. The entire sample removal procedure took less than 5 min. For enzymatic tests, we used the L-Asparagine/L-Glutamine/Ammonia Assay Kit (Megazyme) following the manufacturer's recommendations and using asparagine or glutamine (final concentration 0.6 mM) as substrates. Enzymatic activities were measured by following the disappearance of the NADPH from the buffer along time as an indirect indication of asparagine deamination at 340 nm using a microplate reader (VERSAmax microplate reader, Molecular Devices). Experiments were performed in three independent replicates.

### 4.4 Growth of *S. marcescens* with L-asparaginase

Stocks of Db11 were prepared for growth in RWV as described above in either vertical (simulated microgravity with axis of rotation being perpendicular to the gravity vector) or horizontal (normal gravity RWV-control orientation with the axis of rotation being parallel to the gravity vector) orientations at 37 °C and rotated at 25 rpm (as in Nickerson et al. [19]). One hundred microliters of L-asparaginase (Millipore Sigma) suspended in sterile water was added to the growth media for half of the vessels, and the other half received only 100 μl of the sterile water. Growth of the bacteria was estimated by absorbance at 600 nm and was measured every 3 h. Samples were grown for 24 h total to ensure that stationary phase was reached. The sample removed from the RWV for quantification was replaced with sterile PBS, and any air bubbles were removed before resuming rotation. These experiments were run in three independent replicates.

### 4.5 L-asparaginase treatment

*S. marcescens* in the stationary phase were preserved at -80 °C in 10 mL of 20% glycerol. Concentrations of samples for injections were determined by performing colony counts of *Serratia* in different concentrations of PBS on LB plates. The PBS source without added bacteria was plated as a negative control for contamination. Based on these colony counts, samples were then diluted to 4 × 10^5^ CFU/mL in sterile PBS for injections.

For infections and survival, the Nanoject II (Drummond Scientific) was used with the 32 nL injection volume setting as described previously by our laboratory [11]. Concentrations were adjusted so that each injection was approximately 10 CFU/fly. A total of 180 flies were injected, with 60 total flies assigned to each bacterial treatment group. Control bacteria, simulated microgravity-treated bacteria, or a glycerol sham were then injected into the 30 anesthetized 2-3 day-old males and 30 females (post-eclosion) *D. melanogaster* in the ventral abdomen near the seventh/eighth segment. One hour later, half of those flies (15 per sex) were injected with 32 nL L-asparaginase. After injections, flies were kept at 25 °C on the same standard dextrose food that they were reared on, and survival was monitored every hour for at least 24 h. These experiments were repeated in three independent replicates.

### 4.6 Growth in Davis minimal media supplemented with amino acids

Bacteria were prepared using the protocol previously mentioned, but Davis Minimal Media supplemented with 10 mM of aspartate, glutamine, or a 50/50 mixture of aspartate and glutamine was used in place of LB broth. To quantify growth, 20 μl of sample diluted in 180 μl of PBS was read at an absorbance of A600 every 24 h for 72 h. To keep the volume of the RWV consistent, 20 μl of sterile PBS was placed in each vessel to make up for the volume lost during sampling. These experiments were repeated in three independent replicates. *S. marcescens* in the stationary phase were preserved at -80 °C in 10 mL of 20% glycerol. Concentrations of samples for injections were determined by performing colony counts of *Serratia* in different concentrations of PBS on LB plates. The PBS source without added bacteria were plated as a negative control for contamination. Based on these colony counts, samples were then diluted to 4 × 10^5^ CFU/mL in sterile PBS for injections.

For infections and survival, the Nanoject II (Drummond Scientific) was used with the 32 nL injection volume setting as described previously by our laboratory [11]. Concentrations were adjusted so that each injection was approximately 10 CFU/fly. A total of 30 flies (15 males and 15 females) were infected with each experimental sample, including 30 total flies injected with a glycerol sham. These experiments were repeated in three independent replicates.

### 4.7 Expression analysis of amino acid genes

Samples were diluted to 1 × 10^8^ cells and pelleted, and RNA was extracted from pelleted samples using the RNEasy® Mini Kit (Qiagen). cDNA was generated using the RevertAid First Strand cDNA Synthesis Kit (Thermo Scientific). Relative gene expression levels were quantified using the 7500 Real Time PCR instrument from Applied Biosystems and SYBR™ Green PCR Master Mix (Applied Biosystems). Primers were designed with Primer-Blast, NCBI. Real-time cycler conditions were; 95 °C for 15 min for initial activation step, and 40 cycles of 94 °C for 15 s, 50 °C for 30 s, and 72 °C for 30 s for denaturation, annealing, and extension respectively. Data were normalized to the reference gene *recA*. These experiments were repeated in three independent replicates, including RNA extraction from three independent growth trials and three qPCR runs of each sample (9 total plates). Additionally, each plate (replicate) contained 6 identical wells which were averaged to give a mean C_T_ value of a given gene, ensuring that any pipetting errors were minimized in the data.

### 4.8 Statistical analysis

Statistical analysis was performed in GraphPad Prism 7.0 and JMP Pro 15 (SAS). Graphs were made in GraphPad Prism 7.0 and Excel (version 15.4). For survival analyses, a Cox Proportional Hazards model was used. The Cox Proportional Hazards model allows for the comparison of survival between all groups when there are multiple risk factors or treatments involved. The proportional hazards test generates a ‘risk ratio’ when comparing treatment curves, which can be interpreted in infection experiments as a rate of death. For example, a risk ratio of 2.0 means that one treatment kills the host twice as fast as the treatment it's being compared to. For all growth measurements, a one-way ANOVA was used in combination with a Tukey-Kramer posthoc analysis. For gene expression, expression values are reported as fold change relative to the Davis minimal media values, which were calculated in Excel using the ΔΔ C_t_ method. For all statistical analyses, only results with a statistical significance <0.05 are reported, due to the magnitude of results tables.

## Declarations

### Author contribution statement

Rachel R Gilbert: Conceived and designed the experiments; Performed the experiments; Analyzed and interpreted the data; Wrote the paper.

Nicole Tanenbaum: Performed the experiments; Analyzed and interpreted the data.

Sharmila Bhattacharya: Analyzed and interpreted the data; Contributed reagents, materials, analysis tools, or data; Wrote the paper.

### Funding statement

This work was supported by NASA's Biological and Physical Sciences Division (grant number NNX15AB42G), 10.13039/100006195Ames Research Center Intramural Research Funding, and the International Space Station Research and Development Office (to S.B.), and the NASA Postdoctoral Program (to R.G.).

### Data availability statement

Data will be made available on request.

### Declaration of interests statement

The authors declare no conflict of interest.

### Additional information

No additional information is available for this paper.
